# Safety and Efficacy of Genicular Nerve Radiofrequency Ablation for Management of Painful Total Knee Replacement: A Systematic Review

**DOI:** 10.7759/cureus.19489

**Published:** 2021-11-11

**Authors:** Naga Cheppalli, Amit W Bhandarkar, Senthil Sambandham, Solomon F Oloyede

**Affiliations:** 1 Orthopaedics, University of New Mexico School of Medicine, Albuquerque, USA; 2 Orthopedics, SSM Health St Mary’s Hospital, Centralia, USA; 3 Orthopedics, University of Texas Southwestern Medical Center, Dallas, USA

**Keywords:** genicular nerve, cryoneurolysis, radiofrequency ablation, genicular nerve ablation, knee pain, osteoarthritis of knee, total knee arthroplasty, total knee replacement

## Abstract

Painful total knee replacement (TKR) without an obvious underlying identifiable pathology is not uncommon. Dissatisfaction after TKR can be up to 20%. Different treatment modalities, including non-operative and operative procedures, have been described in the literature. Radiofrequency ablation of genicular nerves (GNRFA) is emerging as a newer treatment modality for painful TKR without an obvious underlying identifiable pathology. Despite a modest number of publications demonstrating the usefulness of GNRFA in managing pain in knee osteoarthritis, the efficacy of GNRFA has not been completely established in the management of residual pain after TKR. This systematic review aimed to analyze all published studies (nine studies) on GNRFA as an option to manage residual pain after TKR. Based on this current systematic review, we noted that GNRFA is a modality to treat post residual pain and patients can anticipate improvement in pain up to three months with minimal complications. This article provides an overview of the currently available knowledge and techniques employed for this procedure, as well as the expected outcome and safety profile of GNRFA in painful TKR.

## Introduction and background

Dissatisfaction after total knee replacement (TKR) can be up to 20% [1]. The most common cause of dissatisfaction after TKR is residual pain in the joint. The reasons for a painful knee TKR include infection, aseptic loosening, instability, malalignment, neuroma, and other rare causes [2]. However, sometimes it is difficult to ascertain any particularly identifiable pathology as a cause of pain despite extensive workup. The initial conservative treatments are oral pain medication, topical therapy, physical therapy, acupuncture, cryotherapy, and lifestyle modification [3]. Periarticular/intra-articular steroid injections are controversial options as there is an increased risk of infection [4,5]. Most surgeons agree that revising TKR is a more invasive option and may result in unpredictable outcomes for the subset of patients whose identifiable cause for post-TKR residual pain is unknown [1].

Nevertheless, minimally invasive modalities can be appealing for managing residual post-TKR knee “residual pain” in patients where no identifiable cause is found. Radiofrequency ablation (RFA) of genicular nerves (GNRFA) is gaining popularity as one of the modalities in managing chronic pain in knee osteoarthritis (KOA) and painful TKR [6-10]. Despite a modest number of publications demonstrating the usefulness of GNRFA in managing pain in KOA, the efficacy of GNRFA has not been completely established in the management of residual pain after TKR. This systematic review aimed to analyze all published studies on GNRFA as an option to manage residual pain after TKR. This article also provides an overview of the currently available knowledge and different techniques employed for this procedure.

## Review

Methodology

Two independent reviewers (NC, AB) performed a search on PubMed using the following MeSH terms: “Total Knee replacement,” “Total Knee arthroplasty,” “Knee pain,” “Genicular nerve ablation,” “Radiofrequency ablation,” “Cryoneurolysis,” “genicular nerve.” We found 34 results. As the number of results was less than anticipated, we also used the keyword “Genicular nerve ablation” on PubMed, Google Scholar, Medline, Cochrane database, Web of Science, CINAHL, and clinical trials.gov through March 31, 2021. We reviewed the results obtained after this search about our inclusion criteria. We also screened all the references from fully retrieved articles not to miss any further relevant studies. Article titles were screened, relevant abstracts were reviewed, and relevant full-text articles were downloaded. We also considered posters presented in the literature. As described below, inclusion criteria and exclusion criteria were rigorously applied to these articles.

Inclusion Criteria

We included all studies in which GNRFA was used to manage residual TKR pain (GNRFA) in this review. Moreover, we included all English-language articles or articles whose translations were available in English.

Exclusion Criteria

We excluded patients who had GNRFA in a knee without an artificial knee, including both total and partial uni knee replacement. We also excluded studies in which RFA was applied to patients with residual TKR pain in the non-genicular nerve distribution. Studies that applied RFA before TKR to manage perioperative pain caused by TKR were also excluded. No cadaveric studies and review articles were included in this review.

Results

**Figure 1:**
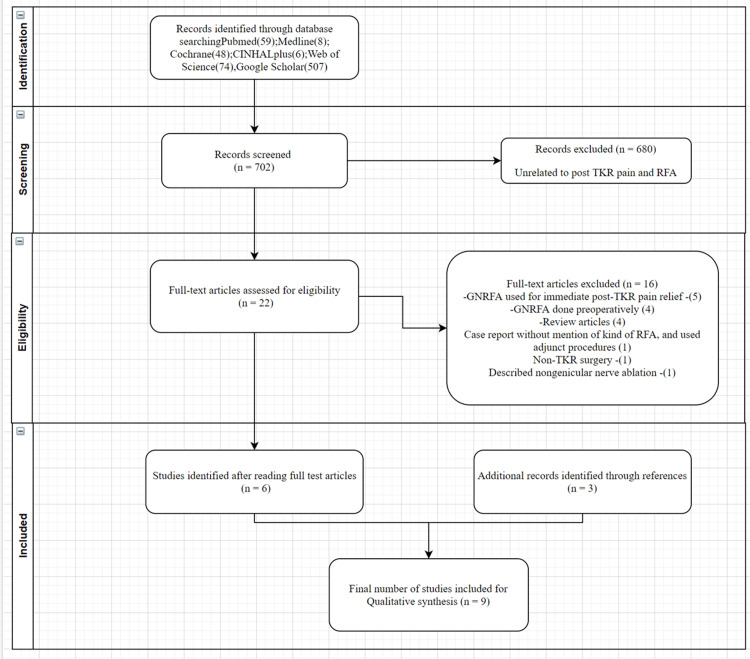
PRISMA flowchart for GNRFA. TKR: total knee replacement; RFA: radiofrequency ablation; GNRFA: radiofrequency ablation of genicular nerves; PRISMA: Preferred Reporting Items for Systematic reviews and Meta-Analyses

We were able to identify 25 relevant articles after a thorough initial review. After reading the full text of the articles, we excluded five articles that discussed using GNRFA for immediate postoperative pain relief [11-15]. Four articles utilizing GNRFA preoperatively for postoperative pain relief were also excluded [16-19]. Four review articles were also excluded [20-23]. One article was a case report of infection after GNRFA. This article included no mention of the kind of RFA utilized and has associated dry needling procedures, which resulted in exclusion from the study [24]. One article discussing GNRFA after another non-TKA knee surgery was also excluded [25]. One of the studies found after searching the references of included articles was subsequently excluded as it described non-genicular nerve supply and used pulsed GNRFA [26]. Finally, a total of nine studies qualified for further analysis, including one randomized, double-blind study, four case series, and four case reports [9,27-34]. We could not obtain the full text of a poster presented at the European Society of Regional Anaesthesia meeting but subsequently included it in the analysis as it contained all required information in the abstract with a substantial number of patients [27] (Figure 1). We gathered available information about the demographics, indications, procedure, pre- and post-procedural visual analog scale (VAS) score, duration of follow-up, functional outcome scores if reported, follow-up duration, and complications related to this procedure (Table 1). We further analyzed the results and the methodological quality of the studies retrieved for this systematic review.

**Table 1:** A summary of included articles. RFA: radiofrequency ablation

Author, Year	Number of patients	Kind of radiofrequency	Pain improvement	Duration of pain relief (in months)	Image guidance	Pre-operative diagnostic blocks used	Local steroid used after the procedure	Evidence level	Country of origin	
										Heated RFA										
Qudsi-Sinclair et al., 2017 [9]	14	RFA 80 degrees for 90 seconds (Cosman Medicals)	Partial improvement	3–6 months	Fluoroscopy	N/A	N/A	Level 1	Spain	
										Sylvester and Goree, 2017 [32]	1	RFA 80 degrees for 120 seconds (Stryker, Kalamazoo)	Improved pain	3 months	Fluoroscopy	N/A	N/A	Case report	USA	
Protzman et al., 2014 [31]	1	RFA 80 degrees for 90 seconds using NT1000 RF generator(NeuroTherm)	Improved pain and function	3 months	Ultrasound	N/A	N/A	Case report	USA											
Yoshimuura et al., 2019 [27]	14	Radiofrequency heated at 80 degrees for 90 seconds	Significant improvement	2 months	Ultrasound	N/A	NA	Case series	Japan	
Leong, 2018 [34]	1	Radiofrequency duration, and temperature not specified	More than 50% improvement	3 months	Fluoroscopy	N/A	40 mg triamcinolone	Case report	Malaysia	
										Gönüllü et al., 2020 [30]	28	Radiofrequency heated at 80 degrees for 90 seconds	Significant pain improvement	3 months	Fluoroscopy	N/A	N/A	Case series. Retrospective study with no controls	Turkey	
Cooled RFA																				
Bellini and Barbieri, 2015 [29]	3	Cooled RF (N/A)	Significant pain improvement in two of three patients	12 months	Fluoroscopy	N/A	N/A	Case series with controls. Sample size too small for adequate comparisons	Italy	
Menzies and Hawkins, 2015 [33]	1*	Cryoablation, (Coolif) temperature N/A, no steroids	Significant improvement	6–9 months	Fluoroscopy	N/A	No steroids	Case report of three nerves	USA	
Alberca et al., 2017 [28]	7	Cryoablation, (Coolif) of three nerves, tip 4 mm for 2.5 minutes at 60 degrees	Improved pain in two patients	12 months	Fluoroscopy	N/A	No steroids	Case series	Spain	

Quality Assessment

The eligible papers were scored according to the National Heart Lung and Blood Institute criteria in quality assessment [35]. One randomized controlled study scored 9 over 14 points, and three case series scored 7 over 9 points [9,28-30]. The rest of the studies were single case reports (Tables 2, 3). Clinical relevance is presented in Table 4 [36]. Six studies scored 5/5, one study scored 3/5, one study scored 4, and one study scored 2 [9,27-34].

**Table 2:** Quality assessment of the included studies: case series. Q1: Was the study question or objective clearly stated? Q2: Was the study population clearly and fully described, including a case definition? Q3: Were the cases consecutive? Q4: Were the subjects comparable? Q5: Was the intervention clearly described? Q6: Were the outcome measures clearly defined, valid, reliable, and implemented consistently across all study participants? Q7: Was the length of follow-up adequate? Q8: Were the statistical methods well-described? Q9: Were the results well-described? Y: yes; N: no

Study	Q1	Q2	Q3	Q4	Q5	Q6	Q7	Q8	Q9	Total score
Bellini and Barbieri [29]	Y	Y	Y	Y	Y	Y	N	Y	N	7
Gönüllü et al. [30]	Y	Y	Y	Y	Y	Y	N	N	Y	7
Alberca et al. [28]	Y	Y	Y	N	Y	Y	Y	Y	N	7

**Table 3:** Quality assessment based on the National Heart Lung and Blood Institute criteria for randomized studies. Q1: Was the study described as randomized, a randomized trial, a randomized clinical trial, or a randomized controlled trial? Q2: Was the method of randomization adequate (i.e., use of randomly generated assignment)? Q3: Was the treatment allocation concealed (so that assignments could not be predicted)? Q4: Were study participants and providers blinded to treatment group assignment? Q5: Were the people assessing the outcomes blinded to the participants’ group assignments? Q6: Were the groups similar at baseline on important characteristics that could affect outcomes (e.g., demographics, risk factors, comorbid conditions)? Q7: Was the overall drop-out rate from the study at the endpoint 20% or lower than the number allocated to treatment? Q8: Was the differential drop-out rate (between treatment groups) at the endpoint 15 percentage points or lower? Q9: Was there high adherence to the intervention protocols for each treatment group? Q10: Were other interventions avoided or similar in the groups (e.g., similar background treatments)? Q11: Were outcomes assessed using valid and reliable measures, implemented consistently across all study participants? Q12: Did the authors report that the sample size was sufficiently large to be able to detect a difference in the main outcome between groups with at least 80% power? Q13: Were the outcomes reported or subgroups analyzed prespecified (i.e., identified before analyses were conducted)? Q14: Were all randomized participants analyzed in the group to which they were originally assigned, i.e., did they use an intention-to-treat analysis? Y: yes; N: no

	Q1	Q2	Q3	Q4	Q5	Q6	Q7	Q8	Q9	Q10	Q11	Q12	Q13	Q14	Total score
Qudsi-Sinclair et al. [9]	Y	Y	Y	N	Y	Y	N	Y	Y	Y	Y	N	N	N	9/14

**Table 4:** Clinical relevance of the studies.

Author	A: Patient	B: Intervention	C: Outcome	D: Effect size	E: Benefit versus harm	Total score	
							Qudsi-Sinclair et al. [9]	+	+	+	+	+	5	
Sylvester and Goree [32]	+	+	+	+	+	5	
Protzman et al. [31]	+	+	+	+	+	5	
Yoshimura et al. [27]	+	+	+	-	U	3	
Leong [34]	+	+	-	+	+	4	
Gönüllü et al. [30]	+	+	+	+	+	5	
Bellini and Barbieri [29].	-	+	+	+	U	5	
Menzies and Hawkins [33]	+	+	+	+	+	5	
Alberca et al. [28]	-	+	+	-	U	2	

We noted several inconsistencies in data presentation, outcomes, protocols, and follow-up. The studies were heterogeneous regarding the outcome measures, duration of follow-up, types of modality, and guidance for RFA used. Therefore, we could not perform a statistical comparison. However, descriptive statistics were reported. The data were normally distributed, as evidenced by the Shapiro Wilk test, and hence, data were analyzed using the parametric test. We obtained pooled data from the above-mentioned selected nine studies (Table 1). The total number of patients included in the analysis is 70. The average age of the patients undergoing the treatment was 68.45 years (data were available in 56 patients), and the male versus female ratio was 1:6. Numeric scale or VAS was used in most of the studies. Preoperative VAS or numerical scale (0-10) was available in 59/70 (84%) patients. The average preoperative score was 7.68. The post-GNRFA VAS score at three months in these patients was 4.2 (59/70 patients). The mean reduction in pain was 3.4 points in the VAS score (55% of reduction). Preoperative VAS was not available for 11 patients [28,29,33]. Only 16/70 (22%) patients had reported pain scores beyond six months. Pain reduction results beyond three months could not be reported as findings were not available for 78% of the patients.

Different functional scores, including Western Ontario and McMaster Universities Osteoarthritis Index (WOMAC), Knee Injury and Osteoarthritis Outcome Score (KOOS), Oxford Knee Score, Short Form 36 (SF-36), were used in these studies, making it difficult to compare the outcomes. Overall, 42/70 (60%) of patients reported WOMAC scores as patient-reported outcome measures (PROM) [27,30]. Yoshimura et al. reported an improvement of 23.2 points in WOMAC after the procedure, whereas Gönüllü et al. reported an improvement of 30.2 points in the WOMAC functional score [27,30]. The overall improvement was approximately 27.86 points in the WOMAC score. Qudsi-Sinclair et al., in their series of 14 patients, reported SF-36 as PROM and noted a 7-point improvement in SF-36 at three months [9].

Three types (conventional, cooled, pulsed) of RFA can be applied for genicular nerve ablation. In this study, after applying exclusion criteria, there were no cases with pulsed GNRFA. Overall, 59/70(84%) patients underwent conventional GNRFA, and 11/70 (16%) patients underwent cooled RFA (Table 1). The mean preoperative VAS score in the conventional RFA group was 7.68 and the postoperative VAS score was 4.27. The score improved by 3.41 points, which accounted for a 55% reduction from preoperative pain at three months. Cooled RFA studies were reported as percentage improvement, but the baseline and postoperative VAS score was not mentioned. Eleven patients underwent cooled RFA. Data were reported for only three patients who had more than 50% improvement in their pain score.

Among 70 patients, 55 (78%) patients underwent the RFA procedure using fluoroscopy, while 15 (22%) received ultrasound guidance for the placement of RFA needles. We attempted to compare the outcomes of these two groups, but sufficient data points and details were not available for a meaningful comparison.

Some authors have recommended using preoperative blocks to predict successful outcomes following GNRFA. On a closer look, only 10 (14%) patients had information about the use of blocks. There is no information available from the rest of the articles. There is a good possibility that the rest of the authors might not have used pre-procedure blocks. However, as this information is not available, we did not draw any conclusions. Three patients received preoperative blocks while seven patients did not receive the blocks [28,31,32,34].

The studies were further analyzed to determine how long after TKR the RFA procedure was performed. This information was available for only 30 (42%) patients. Two authors reported using RFA after six months, while one author reported that RFA was applied in one knee after three years and in another after five years [30,32,33].

Injection of intralesional corticosteroids after GNRFA is practiced by some specialists to reduce post-procedural pain and discomfort. This data was available only for 15 (21%) patients, while the rest of the studies did not mention if steroids were used. Only one patient received corticosteroid injections after the procedure, whereas 14 patients did not receive corticosteroids. All authors used RFA for superomedial, superolateral, and inferior medial genicular nerves. None of the authors reported ablating the other genicular nerve (superior-medial).

None of the studies reported significant complications. Some of the minor complications included pruritus and erythema. One of the studies mentioned increased falls in four patients post-RFA. However, the article was not clear if the patients were from the TKR group. The decrease in proprioceptive input was the probable reason according to the author. We noted one case report of infection after GNRFA which was not included for analysis as it did not mention what kind of RFA and which nerves have been ablated [23]. The patient also underwent a dry needling procedure in addition to GNRFA.

Discussion

TKR is one of the most performed orthopedic procedures for symptomatic KOA. After TKR, it might take three to six months for the post-surgical pain to resolve. Any pain that lasts longer can be classified as post-surgical persistent pain (PSPP) [36,37]. PSPP after TKR is a common cause for dissatisfaction. Its prevalence can range from 16% to 39% at six months and 13.1% to 23% at 12 months [38]. The International Association for the Study of Pain defines chronic pain as pain persisting for three months or longer [39]. Poor mental health, history of chronic pain or pain elsewhere, pain catastrophizing, comorbidities, and high-intensity knee pain are some of the reasons associated with chronic pain after surgery [40]. Sometimes, pain improves a year after TKR [41]. Most surgeons believe that revision TKR surgery performed without proper explanation for the pain can have poor outcomes [6]. Despite the wide prevalence of chronic knee pain after TKA, there is a lack of literature on the best ways to address this problem. In a systematic review, Beswick et al. found inadequate evidence on the effectiveness of prediction and management strategies for chronic pain after TKA [42]. Less invasive options such as intra-articular steroid injections and GNRFA can be attractive options for patients and treating specialists to address post-residual TKR pain. However, some studies claim that intra-articular steroid administration in painful TKA is associated with increased infection risk [4,43]. In this systematic review, we evaluated and analyzed the available evidence and the role of RFA in relieving post-residual pain after TKA.

RFA was first introduced in 1970 to manage chronic pain. It was first successfully used to treat trigeminal neuralgia and later expanded to radiculopathic pain management. Currently, there are three kinds of radiofrequency ablation devices available: conventional (heated) RFA often referred to as RFA, cooled RFA, and pulsed RFA. In RFA, radiofrequency waves are delivered to the target tissue through percutaneously inserted special needles which are insulated in the shaft and have an active tip. The active tip can be 8 mm,10 mm, and 12 mm long, depending upon the lesion area to be targeted. As the radio waves exit through the needle tip, heat is generated through an area of tissue with relatively high resistance. Heat coagulates the local area, denatures local proteins, and results in Wallerian degeneration of the surrounding nerves.

The extent of coagulation is determined by the length of the tip, conduction medium, the needle diameter, the temperature used, and the duration of RFA. It has been hypothesized that RFA preserves the basal lamina of Schwann cells, allowing the possibility of nerve regeneration. RFA also promotes neuromodulation by inhibition of the excitatory C fibers [44,45]. Pulsed RFA was introduced in 1998 as an alternative to conventional RFA.

Although pulsed RFA is similar to heated RFA, as soon as the temperature reaches 41°, the machine cuts off the electricity. It results in less tissue damage and more precise ablation of the nerves, with similar patient outcomes [46]. It has been postulated that pulsed RFA and RFA have similar effects on neuronal conduction but with less irreversible tissue damage. It is a less painful procedure and is associated with a low risk of deafferentation pain. However, the duration of pain relief could be shorter when compared to RFA [47]. Cooled CRFA, introduced in 1996, is another modality of RFA in which chilled saline is passed through the chamber shaft of the needle, with resultant reduction of local temperature, thus limiting excessive tissue damage. Cooled RFA gives the ability to create more neuronal lesion areas, which may improve the effectiveness of the procedure and reduce the number of technical failures. The duration of pain relief could be equivalent to conventional RFA. The destruction of the sensory nerve supply leads to pain relief. The other proposed mechanism of pain reduction is when neuronal tissue is exposed to the electric field, leading to the c-Fos gene expression in lamina I and II of the dorsal horn [48].

GNRFA has become popular after Choi et al. conducted a randomized double-blinded study with conventional RFA and sham control for osteoarthritis chronic knee pain [49]. Since its introduction, multiple studies have shown GNRFA as a successful modality for addressing chronic knee pain from osteoarthritis [50]. Because GNRFA has a proven safety profile for pain management in KOA, multiple authors have used RFA to address post-surgical pain after TKR. This systematic review gathered all the available literature and critically evaluated the quality of evidence for GNRFA to manage chronic residual pain after TKA.

In our review, we noted that there is a lack of high-quality studies on this topic. The available studies are not well structured and do not provide the best evidence [9]. We were able to find only one randomized controlled trial but with a small sample size. Some of the reports have not presented pertinent information such as preoperative VAS score, improvement in functional scores, and procedure details such as pre-procedural blocks and post-procedural steroid administration. There is inconsistent information among articles, for instance, how long after the index procedure GNRFA was utilized. Some of the articles have not mentioned adverse events. We did not find documentation of reduction in opioids in many articles. This review article can help in focusing future researchers on reporting in a more consistent form.

The articles we reviewed showed significant variations in how GNRFA is employed. There were dissimilarities regarding technical steps, the guidance used (ultrasound vs. fluoroscopy), post-procedural use of steroids, the number of nerves ablated, and the type of RFA used (cooled RFA vs. heated RFA vs. pulsed RFA). Due to these heterogeneous ways of applying GNRFA, the complications and outcomes cannot be generalized to GNRFA. We suspect these variations can theoretically lead to different outcomes and complications.

Our systematic review noted a 50-55% reduction in pain. This pain relief can last for three months. Most studies did not report the duration of follow-up beyond three months. The systematic review conducted by Gupta et al. concluded that RFA in KOA (native knee) can cause significant pain relief that can last up to one year with minimal complications [22]. However, RFA evaluation in managing post-residual pain in TKA has not been evaluated for a longer duration such as it has been evaluated in KOA. We recommend that future studies implement a longer follow-up. We cannot anticipate a similar reduction in pain quantity and duration using RFA to manage post-residual pain after TKR. Pain mechanisms can be different in KOA and after TKR. It is also suspected that effective localization of genicular nerves remains unpredictable after correcting the deformity. There might be increased scar tissue around the nerves which may not respond similarly to native tissue. In their series, Alberca et al. noted that only two out of seven patients had a satisfactory outcome [28].

Only one randomized study compared genicular nerve blocks with triamcinolone injection [9]. The study showed that RFA could result in more sustained long-term pain relief than blocks with minimal side effects. However, it did not find any statistically significant difference between blocks using corticosteroids versus RFA. The study concluded that the best results could be anticipated for six months, following which the pain gradually worsens. GNRFA can be performed using either fluoroscopy or ultrasound. Fluoroscopic-guided GNRFA has been validated in multiple studies [49,50]. A prospective study did not show any difference in the outcomes between fluoroscopic-guided and ultrasound-guided genicular nerve blocks [51]. However, ultrasound guidance gives additional details such as the visualization of the tendons and vessels, avoids radiation exposure, and can be more cost-effective. Occasionally, genicular vessels are at risk of injury while performing this procedure [12]. Even though ultrasound-guided RFA appears appealing, there is no convincing evidence to promote this modality over fluoroscopic-guided GNRFA. Ultrasound-guided GNRFA is also technique-dependent. Some authors have used fluoroscopy to initially localize the area of interest and then used ultrasound during the procedure to avoid any tendon damage and vascular lesions.

Some complications such as septic arthritis, inferomedial skin burns, injury to pes anserine tendons, bleeding, and hematoma have been reported with GNRFA in KOA [52-55]. However, these complications have not been reported yet in postoperative TKR cohorts using GNRFA. These complications in the presence of TKA can be devastating, usually requiring either revision surgery or flaps. Clinical relevance assessment of all studies was performed as per the guidelines proposed by Ghogomu et al. [56].

We noted that three of the articles originated from the United States and two from Spain, and the rest from different parts of the world, including Italy, Turkey, and Japan. The first authors of most of the articles were pain and anesthesiology specialists. The first author of one article was from an orthopedic hospital and another from a physical medicine and rehabilitation specialist hospital. None of these articles were published in mainstream orthopedic journals in the United States. Based on this observation, it is possible that this technique is not popular among orthopedic surgeons. We believe that orthopedic surgeons should be well aware of the efficacy of GNRFA as well as the complications involved in this procedure before either performing or referring patients with residual pain after TKR. In our view, this information helps to appropriately counsel patients and set the right expectations before the procedure is performed.

## Conclusions

Although RFA is a well-established technique for pain management, its effectiveness in managing post-residual pain for TKA is not fully established. We highlight the fact that there are no properly conducted studies to recommend this technique for this subset of the population with painful TKA without an obvious cause. Based on published studies, RFA can cause temporary and partial pain relief (50%) that can last for at least three months. Pain reduction beyond three months has not been reported in a majority of articles. No significant complications were reported secondary to this procedure except a case report of infection. Minor complications such as local inflammation have been reported in one case. Because of the limited sample size and inconsistent presentation of the literature, meaningful conclusions could not be derived. Available data do not establish the superiority of one modality of RFA over the other. Further, available studies did not report any major complications even though there are isolated reports of complications. Further studies are needed to generate more robust evidence for the above-mentioned points. We recommend that surgeons use GNRFA with extreme caution in treating post-residual pain after TKA as there is not enough evidence to support the procedure to date.
